# Cost-effectiveness of ravulizumab compared with eculizumab for the treatment of paroxysmal nocturnal hemoglobinuria in the Netherlands

**DOI:** 10.1007/s10198-022-01556-5

**Published:** 2023-01-12

**Authors:** S. W. Quist, A. J. Postma, K. J. Myrén, L. A. de Jong, M. J. Postma

**Affiliations:** 1 Department of Health Sciences, University Medical Center Groningen (UMCG), University of Groningen, Groningen, The Netherlands; 2 Asc Academics, Groningen, The Netherlands; 3 Alexion, AstraZeneca Rare Disease, Stockholm, Sweden; 4 Department of Economics, Econometrics and Finance, University of Groningen, Groningen, The Netherlands

**Keywords:** Ravulizumab, Eculizumab, C5-inhibitors, Hemoglobinuria, Paroxysmal, Cost–benefit analysis, Costs and cost analysis, D61, I19

## Abstract

**Objectives:**

The aim of this study was to evaluate the cost-effectiveness of ravulizumab compared with eculizumab for the treatment of adult patients with paroxysmal nocturnal hemoglobinuria (PNH) in the Netherlands.

**Methods:**

A cost-effectiveness analysis was conducted based on a Markov cohort model simulating the course of patients with PNH with clinical symptom(s) indicative of high disease activity, or who are clinically stable after having been treated with eculizumab for at least the past six months. Costs, quality of life, and the incremental cost-effectiveness ratio (ICER) were estimated over a lifetime horizon from a Dutch societal perspective. Several additional analyses were performed, including a one-way sensitivity analysis, a probabilistic sensitivity analysis, and scenario analysis.

**Results:**

When compared with eculizumab, ravulizumab saves €266,833 and 1.57 quality adjusted life years (QALYs) are gained, resulting in a dominant ICER. Drug costs account for the majority of the total costs in both intervention groups. Cost savings were driven by the difference in total treatment costs of ravulizumab compared with eculizumab caused by the reduced administration frequency, accounting for 98% of the total cost savings. The QALY gain with ravulizumab is largely attributable to the improved quality of life associated with less frequent infusions and BTH events. At a willingness-to-pay threshold of €20,000/QALY, there is a 76.6% probability that ravulizumab would be cost-effective.

**Conclusions:**

The cost reduction and QALY gain associated with the lower rates of BTH and less frequent administration make ravulizumab a cost-saving and clinically beneficial substitute for eculizumab for adults with PNH in the Netherlands.

**Supplementary Information:**

The online version contains supplementary material available at 10.1007/s10198-022-01556-5.

## Introduction

Paroxysmal nocturnal hemoglobinuria (PNH) is a rare, progressive, chronic disease, predominantly diagnosed during young adulthood (30–45 years of age). PNH is characterized by uncontrolled activation of the complement system, leading to chronic intravascular hemolysis and thrombosis. Unless the disease is managed appropriately, life-threatening complications can occur, such as thrombosis, renal insufficiency, and pulmonary hypertension [1–3].

As reported in a 2006 study from England, the incidence of PNH is 0.13 per 100,000 persons, with an estimated 15-year prevalence of 1.59 per 100,000 [4]; using these rates, the PNH incidence and 15-year prevalence among the 17.4 million inhabitants of the Netherlands in 2020 are estimated to be 22 patients and 277 patients, respectively [5]. These estimates include patients with both clinical and subclinical PNH. However, the actual number of diagnosed patients with PNH with high disease activity and who require treatment is lower: in the Netherlands, there are approximately 140 patients diagnosed with PNH, 80–90 of whom require treatment with a complement C5 inhibitor [6, 7]. Though PNH is a chronic disease, it may resolve for some patients, and approximately 5.6–15% of patients with PNH have been found to experience spontaneous remission [8, 9].

Prior to the introduction in 2008 of the first complement C5 inhibitor, eculizumab, PNH treatment in the Netherlands consisted exclusively of supportive symptom management (e.g., transfusions, anticoagulation, and iron supplementation) [3]. Unlike supportive treatment, eculizumab specifically targets the complement C5 protein and thereby prevents complement activation, reducing intravascular hemolysis, thrombosis, and other complications and symptoms in PNH. Data from eculizumab-treated patients show a mean 5-year survival of 95.5%, which is a significant increase compared with supportive care alone, in which around 35% of patients died within five years of diagnosis [10, 11]. Moreover, treatment with eculizumab significantly improves quality of life (QoL) to the point that patients with PNH can live a relatively normal life [12, 13]. Consequently, eculizumab is now the standard of care in the Netherlands for patients with PNH who are experiencing PNH-related intravascular hemolysis, PNH-related thrombosis, and/or related complications [3].

Despite the clinical benefits that eculizumab provides for patients with PNH, the treatment regimen has considerable practical implications for their lives. Patients require infusions of eculizumab every 2 weeks, each with an infusion duration between 25 and 45 min and a total treatment duration between two to four hours [14–16]. Frequent administrations of eculizumab are time-consuming for both patients and caregivers and are considered burdensome by many patients with PNH [16–18]. Additionally, some patients with PNH treated with eculizumab may experience “breakthrough hemolysis” (BTH) either related to incomplete C5 inhibition (IncC5Inhib) by eculizumab or linked to a complement-amplifying condition (CAC) such as an infection, surgery, or pregnancy.

In July 2019, the European Medicines Agency granted marketing authorization to ravulizumab, a new complement inhibitor [19]. Similar to eculizumab, ravulizumab binds to the complement C5 protein; however, while eculizumab is degraded in the endosome, ravulizumab is recycled by the FcRn-receptor and can bind to a second molecule of C5 [20]. Hence, the half-life of ravulizumab is four times longer than that of eculizumab. Therefore, ravulizumab provides immediate, complete, and sustained inhibition of C5 for eight weeks (cf., 2 weeks with eculizumab) [21], and patients treated with ravulizumab only require six or seven infusions per year, instead of 26 when treated with eculizumab [15].

Two pivotal clinical trials, ALXN1210-PNH-301 and ALXN1210-PNH-302 (hereafter referred to as Study 301 and Study 302), have shown that ravulizumab is clinically non-inferior compared with eculizumab in eculizumab-naive patients as well as eculizumab-experienced and stable patients. However, both trials showed numerically (though statistically non-significant) fewer BTH events among ravulizumab-treated patients than among eculizumab-treated patients. Moreover, none of the reported BTH events in the ravulizumab arm were caused by IncC5Inhib [20, 22]. The National Institute for Health and Care Excellence (NICE) and Scottish Medicines Consortium (SMC) have recently recommended ravulizumab as an option for treating PNH. These recommendations are supported by the clinical non-inferiority, the potential reduction in costs, and the increase in QoL associated with ravulizumab, which can be attributed to reduced infusion frequency required [23, 24].

The reduced infusion frequency and lower rate of BTH events associated with ravulizumab treatment are expected to lead to a gain in QoL for patients. In addition, reduced infusion frequency may be linked to a reduction in costs compared with eculizumab in the Dutch clinical practice. The aim of this study was to evaluate the cost-effectiveness of ravulizumab compared with eculizumab in the Netherlands for the treatment of adult patients with PNH with hemolysis, with clinical symptom(s) indicative of high disease activity, or who are clinically stable after having been treated with eculizumab for at least the past six months.

## Methods

A Markov cohort cost-effectiveness model was developed in Microsoft Excel 2016 (Redmond, WA, USA) to determine the cost-effectiveness of ravulizumab as treatment for adult patients with PNH in the Netherlands. The study followed the ISPOR CHEERS checklist and adhered to good reporting practice for health economic evaluations [25].

The model compared ravulizumab with eculizumab, the current standard of care [3]. The difference in QoL between patients treated with ravulizumab and those treated with eculizumab was based on the European Organization for Research and Treatment of Cancer (EORTC) questionnaire scores as measured in the clinical trials, combined with the difference in QoL scores related to the reduced infusion frequency based on results from a discrete choice experiment (DCE) in a sample of the general Dutch population. The analysis was conducted from a Dutch societal perspective. The main outcome was the incremental cost-effectiveness ratio (ICER) of ravulizumab versus eculizumab. The willingness-to-pay (WTP) threshold used was €20,000 per QALY, based on the disease burden calculation (proportional shortfall estimate of 0.07) as recommended by the Dutch guideline for economic evaluations in healthcare [26, 27]. In addition to the base case, several analyses were performed to assess parameter uncertainty and determine the robustness of the model, including a univariate sensitivity analysis, a probabilistic sensitivity analysis (PSA), and scenario analyses. An overview of all the assumptions made in the model can be found in Appendix 1.

### Model structure

**Fig. 1:**
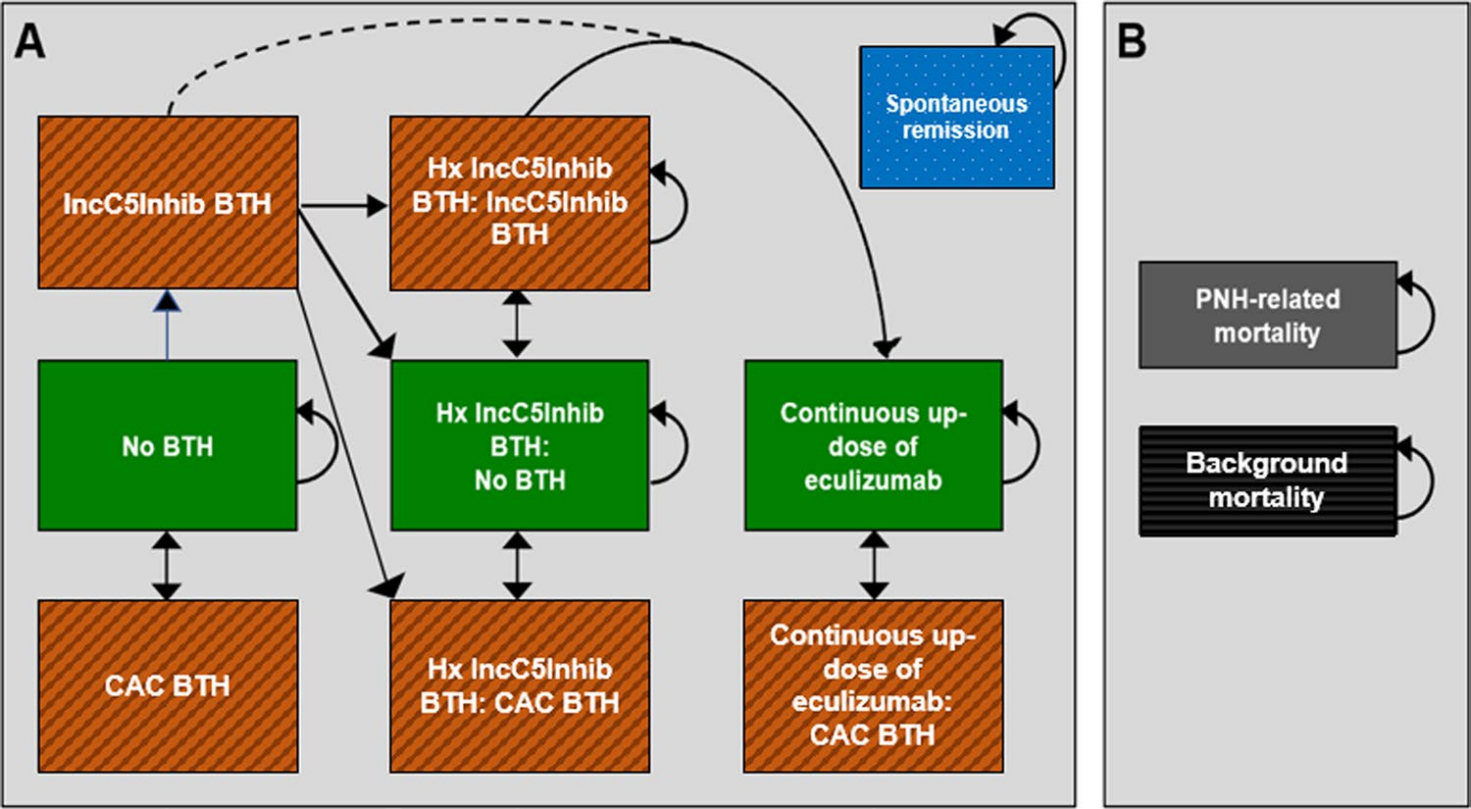
Schematic structure of the cost*-*effectiveness model. *BTH*: breakthrough hemolysis; *CAC* complement-amplifying conditions; *Hx* history of; *IncC5Inhib* incomplete C5 inhibition; *PNH* paroxysmal nocturnal hemoglobinuria. Panel A depicts the living health states, and Panel B depicts the two mortality-related health states. The green-shaded states represent the No BTH health states, and the orange-shaded states represent the BTH health states. Spontaneous remission and background mortality are possible from every living health state. PNH-related mortality is possible from the BTH health states. After the second InC5Inhib BTH, eculizumab patients are permanently up-dosed with one additional vial (from 900 mg to 1200 mg). Continuous up-dosing is only possible for eculizumab-treated patients because the clinical evidence from the pivotal studies has not identified a need to continuously up-dose ravulizumab-treated patients. It is assumed that continuously up-dosed patients cannot experience any further IncC5Inhib-associated BTH events, only CAC-related BTH events

The Markov model comprised the following 11 health states: nine living health states and two death health states (Fig. 1). All patients entered the model in the “No BTH” health state, in which patients were stable on eculizumab or ravulizumab and had no history of BTH caused by IncC5Inhib. Throughout the model, patients could experience BTH events which could be related to either IncC5Inhib or CACs. A history of IncC5Inhib BTH events is a predictive factor for subsequent events. Therefore, patients experiencing a IncC5Inhib BTH move to one of the history IncC5Inhib BTH health states [3]. All eculizumab-treated patients who experienced a second IncC5Inhib BTH event were assumed to have their dose permanently increased by one additional vial (“up-dosing”) and transitioned to the “Continuous up-dose of eculizumab” health state [3]. It was also assumed that patients in the “Continuous up-dose of eculizumab” health state cannot experience any subsequent IncC5Inhib BTH events [20, 22]. Spontaneous remission was possible from any living health state.

The two mortality-related health states were “background mortality” and “PNH-related mortality”. PNH-related mortality was modeled as an excess mortality risk associated with BTH events [28]. Transitions to “background mortality” were possible from any living health state, while transitions to “PNH-related mortality” were only possible from BTH health states.

A two-week cycle length was used, which aligns with the dosing frequency for eculizumab and the visit frequency per study protocol for Studies 301 and 302 [20, 22]. A lifetime horizon was used [27]. The maximum age in the model was assumed to be 101 years.

### Patient cohorts

In the base case analysis, patients were divided into two different starting cohorts: adults who were naive to treatment with a complement-inhibitor (Cohort 1, from Study 301) and adults who were clinically stable on eculizumab at the labeled dosing for at least six months (Cohort 2, from Study 302). Results were calculated for the aggregate population, which was a mix of both cohorts with a distribution of 8.3% and 91.6% of Cohort 1 and 2, respectively (see Appendix 1). This distribution was calculated as the ratio of the number of new patients (Cohort 1) versus the number of prevalent patients (Cohort 2) in the Netherlands [4].

Model patient characteristics reflect the patient populations in Studies 301 and 302 (Table 1) [20, 22]. Mean age and sex of the patients in Studies 301 and 302 were comparable to Dutch patients with PNH, which indicates that Studies 301 and 302 are a valid basis for model inputs [7]. The weight distribution in the model was based on the Dutch general population [29].

**Table 1:** Patient characteristics of studies 301 and 302 [20, 22, 29]

Characteristic	Input value
Cohort 1: Adults who are naive to eculizumab treatment [20]	
Age at first infusion of study drug, mean (SD), *y*	45.5 (15.7)
Female sex, *n* (%)	112 (45.5)
White race, *n* (%)	94 (38.2)
Packed RBC units received within 1 year before study entry, randomization strata, *n* (%)	
0 units	44 (17.9)
1–14 units	157 (63.8)
> 14 units	45 (18.3)
Weight, mean (SD), kg	Age-dependent, based on Dutch Statistics [29]
LDH ratio	
1.5 to < 3 × ULN	34 (13.8)
≥ 3 × ULN	212 (86.2)
LDH at baseline, mean (SD), U/L	1606.4 (752.7)
Cohort 2: Adults who are clinically stable on eculizumab [22]	
Age at first infusion of study drug, mean (SD), *y*	47.7 (14.2)
Female sex, *n* (%)	97 (49.7)
White race, *n* (%	111 (56.9)
Patients who received packed RBCs/whole blood transfusions within 1 year before first dose, *n* (%)	25 (12.8)
Weight, mean (SD), kg	Age-dependent, based on Dutch Statistics [29]
Years on eculizumab before the first study infusion (SD), *y*	5.8 (3.5)
LDH at baseline, mean (SD), U/L	231.6 (49.2)

*LDH* lactate dehydrogenase; *RBC* red blood cell; *SD* standard deviation; *ULN* upper limit of normal

In clinical practice, a third cohort is seen as follows: patients stable on an up-dose of eculizumab. In the scenario analysis, this third cohort was included in the aggregate population, in which 20% of patients treated with eculizumab were up-dosed. This was based on a cohort study in the UK, in which approximately 20% of the patients required a dose in excess of the standard two-weekly 900 mg maintenance dose to achieve and maintain efficacy [30] These patients start their treatment with up-dosed eculizumab treatment (1200 mg) and were, like patients in Cohort 2, assumed to be stable on this dose for at least six months prior to entering the model. The baseline characteristics and utility values of Cohort 3 were, therefore, assumed to be equal to those of Cohort 2.

### Transition probabilities

The transition probabilities for BTH events were based on the 52-week patient visit data from Study 301 for Cohort 1 and Study 302 for Cohort 2 (Table 2). The patient sample was stratified according to history of IncC5Inhib BTH events so that separate transition probabilities could be calculated for initial IncC5Inhib BTH events and subsequent IncC5Inhib BTH events.

**Table 2:** Estimated transition probabilities for BTH-event health states [8, 20, 22]

Transition probability	Cohort 1^a^ [20]		Cohort 2^a^ [22]	
	Eculizumab	Ravulizumab	Eculizumab	Ravulizumab
No BTH to IncC5Inhib BTH	0.0031	–	0.0010	–
No BTH to CAC BTH	0.0054	0.0026	0.0031	0.0001
IncC5Inhib BTH to Hx IncC5Inhib BTH: IncC5Inhib BTH (Second IncC5Inhib BTH)	0.3313	–	0.3321	–
IncC5Inhib BTH to Hx IncC5Inib BTH: CAC BTH	0.0054	–	0.0031	–
IncC5Inhib BTH to Hx IncC5Inhib BTH: No BTH	0.6627		0.6642	
Hx IncC5Inhib BTH: no BTH to Hx IncC5Inhib BTH: IncC5Inhib BTH (Second IncC5Inhib BTH)	0.1420	–	0.3321	–
Hx IncC5Inhib BTH: no BTH to Hx IncC5Inib BTH: CAC BTH	0.0054	–	0.0031	–
Hx IncC5Inhib BTH: IncC5Inhib BTH to Continuous up-dose	0.9994	–	0.9994	–
Continuous up-dose to Continuous up-dose CAC BTH	0.0054		0.0031	–
Any living state to Spontaneous remission [8]	0.0006	0.0006	0.0006	0.0006

*BTH* breakthrough hemolysis; *CAC* complement-amplifying conditions; *Hx* history of; *IncC5Inhib* incomplete C5 inhibition

^a^ The incidence of BTH events was based on 52-week data as there were too few events during the first 26 weeks

Transition matrices were constructed in the following three steps:

1. Patient-visit-level data for Studies 301 and 302 were organized to determine the probability of an initial IncC5Inhib BTH event or a CAC BTH event (including’undetermined’ adjudications) for patients without a history of IncC5Inhib BTH events [20, 22].
2. Based on the subset of data identified in the previous step, a full information maximum likelihood multinomial logit model was estimated to predict the outcome state conditional on the initial state (’No BTH’).
3. The transition equations developed in the last step were used to calculate the mean transition probabilities for each (initial state-follow-up state) pair. Transition probabilities were calculated for both values of the second covariate, which was a binary indicator for whether the patient received ravulizumab or eculizumab in the randomized treatment period (i.e., the first 26 weeks) of the clinical study. Transition matrices for subsequent IncC5Inhib BTH events were determined in the same manner as for the initial IncC5Inhib and CAC BTH event transitions. Details on the transition probability calculations are shown in Appendix 1.

The transition probabilities were extrapolated with a time-dependent change in risk of initial IncC5Inhib BTH for Cohort 1, such that patients in Cohort 1 who have not yet experienced an initial IncC5Inhib BTH event take on the risk observed in Study 302 after 26 weeks of treatment [20, 22]. As IncC5Inhib BTH events were only seen during eculizumab treatment [20, 22], the probability of ravulizumab patients experiencing an IncC5Inhib BTH event was zero. IncC5Inhib BTH events are immediately treated with an earlier than scheduled dose of eculizumab and were, therefore, scaled over the first 2 days of the cycle length in the BTH health state. A CAC BTH event resolves when the underlying condition is resolved (e.g., infection). Therefore, CAC BTH events were assumed to last longer (i.e., a full cycle length) and not to affect the further course of the disease.

The transition probability to spontaneous remission was based on the study by Hillmen et al. (1995) and was equal for all living health states [8]. Background mortality was included to reflect the age-adjusted mortality risk for all patients in the model and was based on the mortality rate for the Dutch general population [31]. The model accounted for the excess risk of PNH mortality by applying the excess mortality rate of 4.8 during the scaled duration of BTH events [32].The model also included packed red blood cell (pRBC) transfusion requirements per treatment arm, with or without a concurrent BTH event, in order to estimate the mean transfusion-related cost and utility impacts (Table 3).

**Table 3:** Transfusion requirement model inputs taken from Studies 301 and 302 [20, 22]

			Study 301 (Cohort 1) [20]		Study 302 (Cohort 2) [22]	
			Eculizumab	Ravulizumab	Eculizumab	Ravulizumab
Patients in no BTH health states	Probability of transfusion in 2-week period	Mean	0.09	0.06	0.02	0.02
		SE	0.01	0.01	0.00	0.00
	Units of pRBC per transfusion	Mean	1.59	1.68	1.57	1.58
		SE	0.07	0.09	0.12	0.12
Patients in BTH health states	Probability of transfusion in 2-week period	Mean	0.30	0.17	0.38	–^a^
		SE	0.10	0.11	0.17	–^a^
	Units of pRBC per transfusion	Mean	1.83	1.50	2.33	–^a^
		SE	0.17	0.50	0.88	–^a^

*BTH* breakthrough hemolysis; *pRBC* packed red blood cells; *SE* standard error

^a^None of the ravulizumab treated patients with BTH received a pRBC transfusion in Study 302

The probabilities of requiring a transfusion in the 2-week horizon of our analysis, as well as the mean number of units of pRBC required, were calculated based on 26-week patient visit-level data from Studies 301 and 302 (Appendix 1) [20, 22].

### Utilities

The utility values were determined in multiple steps as follows:

1. The EORTC Quality of Life Questionnaire (30 items) (EORTC QLQ-C30) outcomes from Studies 301 and 302 were mapped onto EuroQol 5-Dimension 3-Level (EQ-5D-3L) utility values, and results were analyzed with a mixed-effects regression model per health state (Appendix 1, Table S6).
2. The utility increments related to the reduced treatment administration of ravulizumab were calculated based on a DCE in the Netherlands.
3. The per health state transition probabilities were corrected for the Dutch general population utility cap.

The EORTC QLQ-C30 outcomes over the period of 26 weeks were mapped onto Dutch EQ-5D-3L utility data using the direct ordinary least squares regression mapping method by Versteegh et al. (2012) [33]. The effect of using a different mapping method on the QoL outcomes was assessed in a scenario analysis, based on the method of Longworth et al. (2013) [34].

The mixed-effects regression model described the associations between the utility values and BTH events, transfusions, and visit counts. A treatment indicator was omitted to avoid double counting as the utility increment of ravulizumab versus eculizumab was based on the DCE survey. Mixed-effects models were employed to better account for the heterogeneity in the panel data used from the 301 and 302 clinical trials—that is, heterogeneity between individuals in the data and natural fluctuations [20, 22]. The mixed-effects regression model is described in more detail and the outcomes are presented in Appendix 1.

The cost-effectiveness analysis incorporated a health-utility increment for ravulizumab versus eculizumab to reflect the patient benefit of the decreased frequency of administration [17, 35]. The health-related QoL data collected in Studies 301 and 302 did not fully reflect the benefits of the reduced treatment burden of ravulizumab versus eculizumab. In Studies 301 and 302, patients had to visit the study center (per protocol) every two weeks regardless of whether treated with eculizumab or ravulizumab, even if they were not receiving an infusion. Therefore, a DCE was conducted in the Netherlands that determined the utility increment of 6–7 infusions per year of three hours each instead of 26 infusions per year of one hour each. This experiment was previously carried out for the United Kingdom (UK) [36, 37] and was used in the NICE appraisal of ravulizumab for PNH [23]. The design and results of the DCE survey in the Netherlands are summarized in Tables S7–S8. The utility increment of 0.070 found for the ravulizumab treatment arm was implemented in all health states of the ravulizumab treatment arm in the model [38]. The difference in utilities for the treatment arms based on the Studies 301 and 302 was assessed in a scenario analysis, referred to as model “With treatment indicator (tx)”.

An age-specific utility cap for the Dutch general population was included in the modeled health state utilities [39]. The maximum utility value in the spontaneous remission state was used as a reference score, and this score was based on the maximum utility value by age group. The estimates of health utility decrement values relative to the spontaneous remission state for each health state are presented in Table 4, based on the trial data and the utility increment found in the DCE after application of the utility cap. All utilities in the model were discounted at 1.5% per year [27].

**Table 4:** Health utility decrements relative to spontaneous remission applied to age-adjusted, general-population EQ-5D-3L reference utility values [39]

Health state	Cohort 1		Cohort 2	
	Eculizumab	Ravulizumab	Eculizumab	Ravulizumab
No BTH^a^	– 0.097	– 0.026	– 0.071	– 0.002
CAC BTH^a^	– 0.180	– 0.104	– 0.289	– 0.194
IncC5Inhib BTH	– 0.117	N/A	– 0.122	N/A
Hx IncC5Inhib BTH: No BTH	– 0.097	N/A	– 0.071	N/A
Hx IncC5Inhib BTH: IncC5Inhib BTH	– 0.107	N/A	– 0.095	N/A
Hx IncC5Inhib BTH: CAC BTH	– 0.180	N/A	– 0.289	N/A
Continuous up-dose of eculizumab	– 0.097	N/A	– 0.071	N/A
Continuous up-dose of eculizumab: CAC BTH	– 0.180	N/A	– 0.289	N/A
Spontaneous remission	Reference (mean age-adjusted, Dutch general-population health utility)			
Age range	Utility reference values Dutch general population–TTO [38]			
35–44	0.935			
45–54	0.890			
55–64	0.890			
65–74	0.886			

*BTH* breakthrough hemolysis; *CAC* complement-amplifying condition; ***c****TTO* composite time trade-off; *Hx* history of; *N/A* not applicable

^a^The linear visit-count term is used to calculate a representative health-utility estimate for the trial period, reflecting the mean of utility at visits 1 and 6. For study 301 (Cohort 1), the coefficient on this term is positive (0.02), aligning with the expectation that health utility for patients on both treatment arms might improve over time, due to the marked reduction in hemolysis following the start of both treatments. In study 302 (Cohort 2), patients’ hemolysis was already generally controlled at baseline, as this trial population consisted of patients who are clinically stable after having been treated with eculizumab for at least the past six months. The visit-count term is small in magnitude (0.00) as a result, and therefore does not substantially affect modeled utilities. This explains any differences found for the utilities between Cohort 1 and Cohort 2

### Costs

Both direct medical and societal costs were included in the model, as summarized in Table 5. Societal costs included costs for patients and family or costs in other sectors (i.e., travel costs and productivity losses). All costs were adjusted to Dutch values and updated to the year 2020 [40]. All costs were discounted at a rate of 4% [27].

**Table 5:** Unit costs included in the model [3, 14–16, 40, 41, 45]

Unit	Unit costs	Source
Drug and administration costs		
300 mg vial eculizumab	€4316^a^	Z-index eculizumab, EPAR eculizumab [14]
300 mg vial ravulizumab	€4709^a^	Z-index ravulizumab, EPAR ravulizumab [15]
Administration costs eculizumab (per injection)	€182.29	NZa tariffs^b^ [40]
Administration costs ravulizumab (per injection)	€334.22	NZa tariffs^c^ [40]
Medical costs		
General ward admission (day)	€678.99	Dutch cost research manual^d^ [42]
Intensive care admission (day)	€2151	Dutch cost research manual^e^ [42]
Dialysis period of 2 weeks (7 sessions)	€5955	NZa tariffs^fg^ [40]
Hematology specialist visit	€140.92	PNH guideline [3] dutch cost research manual^h^ [42]
Weighted average medical costs per IncC5Inhib BTH event	€502.48	
Weighted average medical costs per CAC BTH event	€869.14	
Unit cost of transfusion administration	€225.26	Dutch cost research manual^j^ [42]
Unit cost of packed red blood cells	€230.60	Dutch cost research manual^j^ [42]
Serotypes ACWY vaccine	€46.43	Medicijnkosten.nl^k^ [43]
Serotype B vaccine	€83.11	Medicijnkosten.nl^l^ [43]
Ciprofloxacin 14 days	€12.88	Medicijnkosten.nl^m^ [43]
Total costs of the meningococcal infection prevention	€142.42	
Societal costs		
Travel costs (per km)	€0.19	Dutch cost research manual [42]
Parking costs	€3.00	Dutch cost research manual [42]
Patients traveling by car	50%	Assumption
Travel distance BTH event and transfusion	14	Dutch cost research manual [42]
Travel distance in-hospital administration	147	Assumption
Productivity loss eculizumab administration	€81.82 (per 4 h)	Levy et al. 2020, dutch statistics, dutch cost research manual [16, 42, 44]
Productivity loss ravulizumab administration	€112.51 (per 5.5 h)	Levy et al. 2020, dutch statistics, dutch cost research Manual [16, 42, 44]
Productivity loss general ward admission	€2811 (per 120 h)	Assumption, dutch cost research manual [42]
Productivity loss intensive care admission	€15,930 (per 680 h)	Assumed full friction period (85 days), dutch cost research manual^n^ [42]
Productivity loss dialysis period of 2 weeks	€749.65 (per 28 h)	Assumed to be half a day of lost productivity per session
Productivity loss hematology specialist visit	€187.41 (per 8 h)	Assumption, dutch cost research manual [42]

*BTH* breakthrough hemolysis; *CAC* complement amplifying condition; *IncC5Inhib* incomplete C5 inhibition; *EPAR* European public assessment report; *SAGM* Saline, Adenine, Glucose, and Mannitol

^a^Including value added tax (VAT)

^b^Code: 193,387: “Eculizumab, dosage form infusion fluid, per unit of 1 mg used for indications included with this substance name in the NZa Performance and tariff table add-on”

^c^Code: 193,384: “Galsulfase, administration form infusion liquid, per unit of 1 mg used for indications included under this substance name in the NZa Performance and tariff table for add-on medicines.”

^d^Based on a nursing day for haemato-oncology (incl. Diagnostics and medication)

^e^Based on an intensive care nursing day (incl. diagnostics and medication)

^f^Based on code 140,301,025: Filtering the blood by an artificial kidney (dialysis) with 4 or 5 dialyses per week in sudden kidney failure, second week

^g^Based on code 140,301,009: Filtering the blood by an artificial kidney (dialysis) with 1 to a maximum of 3 dialyses per week in sudden kidney failure, first week

^h^Based on polyclinic visits for hemato-oncology

^i^Based on a nurse patient day

^j^Based on erythrocytes in SAGM

^k^Based on the average cost of Menveo and Nimenrix

^l^Based on the average cost of Bexsero and Trumenba

^m^Based on 750 mg twice daily for 14 days

^n^Assumed as a full friction period

Drug costs were based on the treatment regimen and list prices for the interventions. Eculizumab treatment was administered weekly for 5 weeks (the initial phase), then every two weeks from week five onwards (the maintenance phase). The dose was 600 mg during the initial phase and 900 mg during the maintenance phase [14]. Patients who experienced their second IncC5Inhib BTH event were up-dosed from 900 mg to 1200 mg eculizumab per dose. Following an initial loading dose, maintenance doses for ravulizumab treatment were administered every eight weeks [14, 15]. For ravulizumab, the initial and maintenance phase doses are weight-based; therefore, these depended on the modeled weight per age in the Netherlands (Appendix 1) [15, 29].

Eculizumab was administered to patients as an intravenous infusion over 25–45 min. Ravulizumab was administered to patients as an intravenous infusion over a minimum of 102–114 min for the loading dose and a minimum of 120–140 min for the maintenance dose. The infusion costs for eculizumab were based on the 2016 Dutch Healthcare Authority (NZa) add-on tariff for eculizumab infusion [41]. The administration costs for ravulizumab were based on the administration tariff for galsulfase, which has a comparable infusion duration (120–140 min). Notably, a higher concentration formulation of ravulizumab (100 mg/ml instead of 10 mg/ml) was also recently approved by the EMA and is currently available in the Netherlands. For the 100 mg/ml formulation, the infusion duration approximates that of eculizumab (25–75 min); hence, the administration costs are similar. This is explored in a scenario analysis [15].

The modeled medical resource utilization associated with a BTH event (Appendix 1 is based on a cost burden study of BTH in patients with PNH [42]. The estimated unit costs were based on tariffs from the Dutch Cost Research Manual and the NZa [41, 43]. The two-weekly costs of the BTH events were calculated by multiplying the resource utilization estimates with their corresponding unit costs.

A pRBC transfusion requires pre-transfusion testing, including the identification of ABO blood types, Rh factor, and other antigens, antibody screening, and cross-matching [3]. These pre-transfusion procedures, along with the administration of the transfusion itself, were costed based on the costs associated with a nurse–patient day in the Dutch Cost Research Manual [43].

Complement inhibitor therapy may increase the risk of meningococcal infection, and it is recommended to vaccinate patients against *N. meningitidis* serogroups A, B, C, W135, and Y on the first day of eculizumab or ravulizumab treatment to minimize this risk [3]. The costs of the meningococcal A, C, W135, Y combination vaccine and the monovalent B vaccine were based on the Dutch list price and only taken into account for treatment-naive patients (Cohort 1) [44].

Travel costs were considered for infusions of eculizumab or ravulizumab, transfusions, and BTH events. Transfusions and treatment of BTH events (general ward admission, intensive care admission, and hematologist visit) were assumed to require one-time travel (back and forth) per visit. The travel costs of dialysis were based on seven sessions (i.e., seven visits) to a dialysis center [41]. It was assumed that 50% of patients used personal transport. For those patients, parking costs were also considered.

Productivity losses resulting from both eculizumab and ravulizumab infusions and BTH events were taken into account. The costs were based on the cost of productivity per hour, corrected for the average labor participation rate. The productivity losses for eculizumab and ravulizumab administration included travel time to the clinic, infusion time for loading and maintenance doses, and recovery time. The productivity losses for transfusion and BTH events consisted of travel time to the clinic, hospital stay, and recovery time [16]. It was assumed that the 50% of patients who travelled to and from the hospital by car required the assistance of a caregiver. To account for the productivity losses of the caregiver, the productivity losses calculated for the patients were multiplied by 1.5.

### Sensitivity and scenario analyses

A univariate sensitivity analysis was conducted to assess the impact of varying certain input variables on the ICER. In the PSA, the input values were simultaneously varied over their confidence intervals (CIs) for a total of 1,000 simulations. The parameters in the analysis were varied by their respective distributions (Normal, Beta or Gamma) using the deterministic values and standard errors. We assumed the standard error to be 25% from the mean when the standard error or 95% CI was not available. For the univariate sensitivity analysis, the lower and upper bounds were set at 2.5% and 97.5%, respectively. Appendix 1 summarizes all the variables and their respective CIs and distributions. The scenario analyses conducted for the cost-effectiveness analysis comprised 25 different scenarios (Appendix 1).

## Results

### Base case analysis

The aggregate population for the base case analysis comprised a total population of 84 patients as follows: 7 complement-inhibitor naïve patients and 77 eculizumab-experienced patients. The base case results show that patients treated with ravulizumab incurred €5,626,489 in total costs, whereas patients treated with eculizumab incurred €5,893,322 in total costs. Additionally, a total of 24.85 QALYs were gained in the ravulizumab cohort versus 23.28 QALYs gained in the eculizumab cohort (Table 6). Hence, ravulizumab saves a total of €266,833 while gaining 1.57 QALYs over a lifetime compared with eculizumab, resulting in a dominant ICER.

**Table 6:** Increment costs, effects, and ICER per patient in cohorts 1 and 2 and the aggregate population for the base case analysis

	Ravulizumab	Eculizumab	Difference
*Cohort 1*			
Drug and administration costs	€5,518,020	€5,811,145	€– 293,125
Medical costs	€14,942	€23,270	– €8329
Societal costs	€102,588	€107,204	€– 4617
Total costs	€5,635,549	€5,941,620	€– 306,071
Total effects (QALY)	24.30	22.70	1.60
ICER (€/QALY)			Dominant
*Cohort 2*			
Drug and administration costs	€5,521,074	€5,778,703	€– 257,629
Medical costs	€5484	€6448	€– 964
Societal costs	€99,107	€103,780	€– 4673
Total costs	€5,625,665	€5,888,931	€– 263,266
Total effects (QALY)	24.90	23.33	1.57
ICER (€/QALY)			Dominant
*Aggregate population*			
Drug and administration costs	€5,520,820	€5,781,406	€– 260,587
Medical costs	€6272	€7,850	€– 1578
Societal costs	€ 99,397	€104,066	€– 4699
Total costs	€5,626,489	€5,893,322	€– 266,833
Total effects (QALY)	24.85	23.28	1.57
ICER (€/QALY)			Dominant

*ICER* incremental cost-effectiveness ratio; *QALY* quality-adjusted life year

The difference in QALY gain between the two interventions is predominantly due to the lower administration frequency associated with ravulizumab as well as the absence of IncC5Inhib BTH events following ravulizumab administration. Treatment costs (drug and administration costs) are the highest contributors to the total costs in both the ravulizumab and eculizumab groups with €5,520,820 and € 5,781,406, respectively, with a difference of €260,587. This accounted for 98% of the total cost reduction. The other 2% of cost savings were due to medical costs related to the prevented BTH events (€– 1578) and societal costs (-€4699).

## Sensitivity and scenario analyses

### Univariate sensitivity analysis

**Fig. 2:**
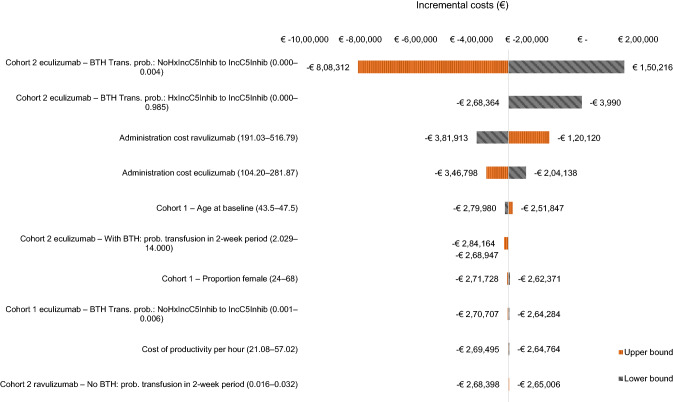
Tornado diagram illustrating the impact on the incremental costs from the univariate sensitivity analysis for the aggregate population. *BTH* breakthrough hemolysis; *Hx* history; *IncC5Inhib* incomplete C5 inhibition; *trans prob* transition probability

**Fig. 3:**
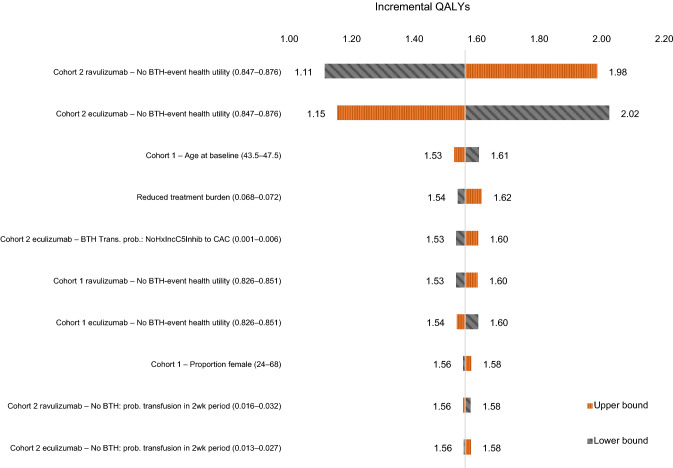
Tornado diagram illustrating the impact on the incremental QALYs from the univariate sensitivity analysis for the aggregate population. *BTH* breakthrough hemolysis; *CAC* complement-amplifying condition; *Hx* history; *IncC5Inhib* incomplete C5 inhibition; *trans prob* transition probability

The results of the univariate sensitivity analyses for the aggregate population are presented as tornado diagrams in Figs. 2 and 3. Notably, the input with the greatest impact on the incremental costs for the aggregate population was drug costs—varying the drug costs for eculizumab resulted in €– 3,345,061 for the upper bound and €2,147,285 for the lower bound, and €– 2,516,968 for the lower bound and €2,602,256 for the upper bound for ravulizumab. Because including these parameters would marginalize the impact of the other parameters in the tornado diagram, they have been left out for presentation purposes (Figs. 2 and 3). Excluding the drug costs for eculizumab and ravulizumab, the tornado diagram for the aggregate population reveals that the inputs with the greatest impact on incremental costs are two transition probabilities for patients in Cohort 2 treated with eculizumab — from the “No BTH” health state to the “IncC5Inhib BTH” health state and from the “Hx IncC5Inhib BTH: No BTH” health state to the “Hx_IncC5Inhib BTH: IncC5Inhib BTH” health state — followed by the administration cost of both drugs. The health utility for patients without BTH for both treatments and baseline age have the greatest impact on the incremental QALYs. The univariate sensitivity analyses for Cohort 1 and Cohort 2 showed similar results to each other (Appendix 2).

### Probabilistic sensitivity analysis

**Fig. 4:**
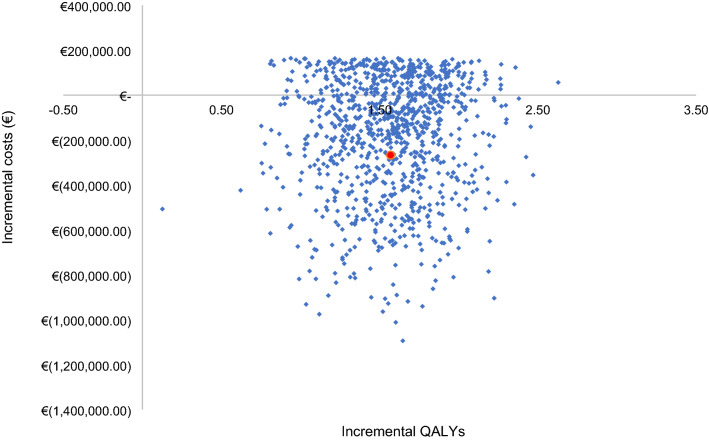
Cost-effectiveness plane for the aggregate population. The red mark reflects the base case ICER. *QALYs* quality-adjusted life years

**Fig. 5:**
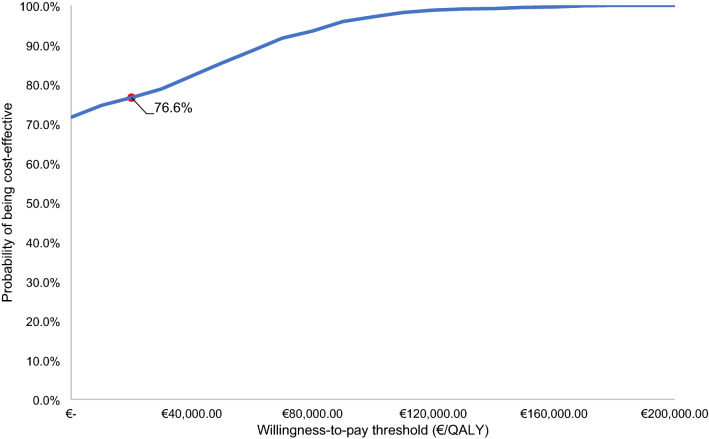
Cost-effectiveness acceptability curve for the aggregate population. The red mark represents the probability of being cost-effective at a WTP-threshold of €20,000 per QALY

The results of the PSA for the aggregate population demonstrate the outcomes for the ICER represented on a cost-effectiveness plane (Fig. 4) and a cost-effectiveness acceptability curve (CEAC) (Fig. 5). The plot on the cost-effectiveness plane shows that the lower and upper bounds (95% CI) of the incremental costs are –€784,916 and €152,875, respectively, with a mean of –€173,252. The lower and upper bounds (95% CI) of the incremental QALYs are 0.92 and 2.19, respectively, with a mean of 1.56. The uncertainty is skewed in a cost-saving direction due to the BTH transition probabilities, which are the critical uncertainty drivers in the model. These transition probabilities were sampled via a Beta distribution. In particular, the transitions of patients with no history of BTH to IncC5Inhib BTH seems to be the cause of the skewed uncertainty, as these are sampled via a beta distribution and have a value close to 0. The cost-effectiveness planes for Cohort 1 and Cohort 2 are presented in Appendix 2. The CEAC plots the probability of cost-effectiveness with a WTP threshold of €20,000/QALY for the aggregate population, which is the lowest threshold used in the Netherlands. The probability of ravulizumab being cost-effective at a WTP of €20,000/QALY is 76.6%. The CEACs for Cohort 1 and Cohort 2 (Appendix 2) shows that the probability of ravulizumab being cost-effective at WTP of €20,000/QALY is 91.2% for Cohort 1 and 75.3% for Cohort 2.

### Scenario analyses

In the aggregate population, the two scenarios with the most impact on the incremental costs are excluding the option of up-dosing eculizumab-treated patients and excluding discounting, resulting in a difference from the base case of €117,874 and –€766,356, respectively (Table 7). The two scenarios with the most impact on the incremental QALYs are basing the utility increment on clinical trial results (0.61 QALYs gained) and setting the time horizon to 10 years (0.60 QALYs gained). We conducted a scenario analysis to assess the effect of including 20% of eculizumab patients as up-dosed (Cohort 3) on the outcomes. This scenario showed that when up-dosing of eculizumab is considered, ravulizumab is even more cost saving (€-529,600 over lifetime).

**Table 7:** Results of scenario analyses for the aggregate population

Subject	Incremental costs	Incremental QALYs	ICER (€/QALY)
Base case	€– 266,833	1.57	Dominant
Time horizon was set to 10 years	€3958	0.61	€6.514
Time horizon was set to 20 years	€– 92,238	1.04	Dominant
Discount rate benefits and costs were set to 0%	€– 766,356	2.02	Dominant
Discount rate benefits and costs were set to 6%	€– 161,288	0.88	Dominant
IncC5Inhib BTH duration (days) were set to 1 day	€– 266,919	1.57	Dominant
IncC5Inhib BTH duration (days) were set to 3 days	€– 266,748	1.57	Dominant
IncC5Inhib BTH duration (days) were set to 7 days	€– 266,405	1.57	Dominant
Formulation ravulizumab was set to 100 mg/mL	€– 388,929	1.57	Dominant
Cohort 3 was included in the aggregate population (20% patients started in Cohort 3 [45])	€– 529,600	1.57	Dominant
Utility increment of ravulizumab vs, eculizumab was calculated from studies 301 and 302	€– 266,833	0.60	Dominant
The general health-utility cap was excluded	€– 266,833	1.57	Dominant
EORTC to EQ-5D-3L mapping based on Longworth (2013)	€– 266,833	1.57	Dominant
HRQoL regression analysis based on pooled population	€– 266,833	1.56	Dominant
No up-dosing of eculizumab happened after IncC5Inhib BTH events^a^	€ 117,874	1.71	€68,967
Continuous up-dosing of eculizumab after first IncC5Inhib BTH event^a^	€– 270,483	1.57	Dominant
BTH excess mortality (HR) vs. background was set to 1.20	€– 272,361	1.54	Dominant
BTH excess mortality (HR) vs. background was set to 0	€– 274,212	1.53	Dominant
Percentage receiving Alexion-funded homecare was set to 100%	€– 389,062	1.57	Dominant
Extrapolation of initial IncC5Inhib BTH risk after 26 weeks was excluded	€– 303,649	1.57	Dominant
Persistence of IncC5Inhib BTH was user defined 25%	€– 265,888	1.57	Dominant
Persistence of IncC5Inhib BTH was user defined 75%	€– 266,662	1.57	Dominant
Days in 2-week cycle with IncC5Inhib BTH symptoms was set to 4	€– 269,230	1.57	Dominant
CAC BTH up-dosing was included^a^	€– 276,709	1.53	Dominant
Healthcare payer's perspective was used and societal costs were excluded	€– 262,165	1.57	Dominant
Spontaneous remission was excluded	€– 404,733	2.06	Dominant

*BTH* breakthrough hemolysis; *CAC* complement amplifying condition; *DCE* discrete choice experiment; *HR* hazard ratio; HRQoL: health related quality of life; *Hx* history of; *ICER* incremental cost-effectiveness ratio; *IncC5Inhib* incomplete C5 inhibition; *NA* not applicable; *QALYs* quality-adjusted life years

^a^ Patients who experiencing a second IncC5Inhib BTH event, were assumed to have their dose permanently increased by one additional vial (“up-dosing”)

## Discussion

The introduction of complement-inhibitor eculizumab in 2008 has substantially changed the prognosis for patients with PNH and is currently the standard of care [3]. However, patients on eculizumab require an infusion every two weeks, and this high administration frequency has a considerable impact on the lives of patients [17]. In contrast, ravulizumab is administered every eight weeks, thereby reducing the burden of treatment. Moreover, there have been no IncC5Inhib BTH events reported with ravulizumab use in any of the clinical trials (including the ongoing extension phases); this absence of events is likely as a result of the longer half-life of ravulizumab [21, 46]. In addition to patients, less frequent treatment requirements and a reduction in BTH incidence are also beneficial for physicians and caregivers. A cost-effectiveness analysis was used to capture all health and economic benefits associated with the use of ravulizumab compared with eculizumab, which can be used to inform decision making.

Our results show that ravulizumab is cost-saving (€– 266,833 per patient) and more effective (1.57 QALYs gained per patient) compared with eculizumab from a Dutch societal perspective. This means that from a healthcare as well as an economic perspective ravulizumab would be a good alternative to eculizumab as a treatment for adult patients with PNH in the Netherlands. Cost savings were driven by the difference in total treatment costs of ravulizumab compared with eculizumab, accounting for 98% of the total cost savings. The gain in QALYs is predominantly caused by the lower administration frequency associated with ravulizumab and the absence of IncC5Inhib BTH events after initiation of ravulizumab treatment.

Several assumptions were required to optimally reflect everyday clinical practice while using the currently available data, and these assumptions can have a considerable impact on the overall structure of the cost-effectiveness analysis. When developing the model, for example, a lack of long-term data meant that it was necessary to extrapolate the 26-week trial data over a lifetime horizon. It was assumed that the trial results after 26 weeks would remain constant over the lifetime horizon of the model, in terms of the incidence of meeting transfusion requirements, probabilities of BTH events, and QoL outcomes. The univariate sensitivity analysis showed that the transition probability for IncC5Inhib BTH had a major effect on the outcome, which indicates that assuming that the probabilities stay equal over time may impact our outcomes. However, the 52-week results of Studies 301 and 302 support our assumption and demonstrate that the non-inferior yet higher efficacy and QoL outcomes of ravulizumab in the first 26 weeks were maintained for the following 26 weeks [47, 48]. More recent data shows that ravulizumab remains well tolerated up to two years after initiating treatment [49]. In addition, the number of InC5Inhib BTH events in our analysis seems to correspond to values found in the literature: In our study, 6–8% of eculizumab patients experience an InC5Inhib BTH event within the first 2.5 years of treatment. This is slightly lower than the 10% previously reported in the literature [50]. Nevertheless, collecting long-term data is important for assessing whether the effect of ravulizumab on IncC5Inhib BTH can be maintained over the years.

Although our analysis represents the Dutch clinical setting as accurately as possible, limited data availability affected our ability to comprise all aspects of complement inhibitor treatment in the Netherlands, and there are several limitations that should be taken into account when interpreting results. First, a lack of data on the number of at-home infusions and their associated costs meant that at-home administration was not implemented in our model. A Dutch study comparing at-home and in-hospital administration of trastuzumab showed that the costs are comparable overall for the two settings [51]. Although the healthcare-related costs of at-home administration were higher compared with in-hospital administration, societal costs were lower due to reduced patient travel costs and productivity losses. Similarly, a study in the United States showed that PNH productivity losses may be saved with at-home administration, while healthcare costs are expected to increase [52]. Thus, the exclusion of at-home administration costs may have impacted the incremental costs. Nevertheless, the scenario analysis showed that ravulizumab was still cost saving when societal costs were excluded (i.e., the healthcare payer’s perspective).

In practice, some patients may require a permanent increase in the dose of eculizumab due to an increased risk of IncC5Inhib BTH, but this was not accounted for in our base case analysis. A study in Leeds, UK, reported that around 20% of patients treated with eculizumab require a dose in excess of the standard two-weekly 900 mg maintenance dose to achieve and maintain efficacy [30]. We conducted a scenario analysis to assess the effect of including 20% of eculizumab patients as up-dosed (Cohort 3) on the outcomes. The results showed that including Cohort 3 in the aggregate population leads to even higher cost-saving outcomes compared with the base case analysis, which is mainly due to the higher drug costs associated with up-dosing. Therefore, a switch to ravulizumab for patients who require up-dosing of eculizumab may be beneficial from both a clinical and a cost-effectiveness perspective.

The conservative method used to estimate mortality risks is also a potential limitation of our study resulting from a lack of data. Study 301 and Study 302 were trials with relatively small patient populations and, as a result, limited data regarding mortality risks. It was assumed that, outside BTH events, the mortality of patients with PNH was equal to the mortality of the general Dutch population and therefore health-state independent. BTH events were assumed to temporarily increase the mortality risk of patients with PNH. As only limited data were available, we based the excess mortality on a study which included only patients with PNH who were not treated with a C5-inhibititor [32]. However, though the estimate may be conservative, the scenario analysis showed that applying a lower excess mortality risk estimate during BTH events did not lead to significantly different outcomes.

Economic evaluations of healthcare interventions clinical trials often have differing recommendations for QoL data collection and analysis, and methods for converting one form of data to another can introduce uncertainty into the model. In study 301 and 302, QoL was assessed using an EORTC instrument because this questionnaire is considered the most adequate measure of QoL in PNH [53]. However, the Dutch guideline for economic evaluations in healthcare recommends that EQ-5D data are used in cost-effectiveness analyses, and mapping methods are recommended if EQ-5D data is not available [34, 43]. For this reason, we mapped the EORTC data to Dutch EQ-5D-3L utility values using the direct mapping method by Versteegh et al. (2012) [33]. Though the use of mapping methods leads to uncertainty in the model, we assessed the impact of using a different mapping method in the scenario analysis (based on Longworth et al. (2013) [34]), and this analysis showed a difference in incremental QALYs of only 0.01, with ravulizumab remaining dominant. Moreover, the PSA solely found positive incremental QALYs with a 95% CI of 1.16 to 2.04, suggesting a high level of robustness in these results.

An analysis assessing patient preferences has shown that patients with PNH who had been treated with both ravulizumab and eculizumab prefer a lower frequency of infusions (once every eight-weeks rather than once every two weeks), and the improved overall QoL they experienced as a result [54]. Study 301 and Study 302 could not show this improvement in QoL because, regardless of the infusion regimen, patients were required to attend the study center every two weeks [20, 22]. Therefore, the QoL data for ravulizumab patients retrieved from Study 301 and Study 302 were corrected with a utility increment based on a DCE performed in the Netherlands [38]. Though the DCE used a scientific approach and has been accepted by NICE for demonstrating the effect of infusion frequency on patient QoL, the use of different sources to establish patient QoL is not ideal and may have impaired the reliability of the input data. For example, DCE participants were from the general public and may have underestimated the impact of a lifelong bi-weekly dosing routine, resulting in an underestimation for the utility increment. However, the scenario analysis established that calculating the utility increment of ravulizumab versus eculizumab based on the study data instead led to lower incremental QALYs (0.60 vs. 1.57), but ravulizumab still improved QoL and was dominant over eculizumab.

In addition, ravulizumab has recently been made available at a concentration ten times the standard dose (i.e., 100 mg/mL instead of 10 mg/mL), making it possible to reduce infusion duration from 120–140 min to 25–75 min [19]. This new infusion time approximates that of eculizumab; therefore, the DCE utility increment may underestimate the QoL difference with this new dose. The effect of the new formulation of ravulizumab was also examined in a scenario analysis. The scenario analysis showed that the new 100 mg/mL formulation is more cost-saving than the 10 mg/mL formulation, mainly due to reduced administration costs and productivity losses. However, the scenario does not consider the beneficial effects of a reduced infusion duration on the QoL of patients with PNH.

This is the first study to analyze the cost-effectiveness of ravulizumab versus eculizumab for patients with PNH in the Dutch setting, following the Dutch guidelines. The model provides a reliable representation of the Dutch setting because it includes societal costs, and it is fully populated with Dutch data, Dutch costs inputs, Dutch value sets for the utility mapping, and a DCE survey specific for the Dutch population. In addition, this is the first study that estimates the cost-effectiveness of ravulizumab versus eculizumab from a societal perspective. A societal perspective is relevant for treating chronic PNH with C5-inhibitors because a reduced infusion frequency brings advantages for patient and society that are only captured when societal costs are included (i.e., reduced travel costs and productivity losses). The inclusion of both treatment-naïve patients and eculizumab-experienced patients is an additional strength of our model, as it provides direct comparative data for these patient populations.

Our results show that ravulizumab is likely to be cost saving compared with eculizumab. However, future research is needed to determine whether the real-world long-term effects of ravulizumab support the assumption of extrapolating 26-week efficacy data to a lifetime horizon. In particular, the absence of BTH events and lack of required up-dosing among ravulizumab patients has not yet been demonstrated in the long-term and has a heavy influence on the model outcomes. Moreover, more data are needed to support the effect of the reduced administration frequency on QoL in patients with PNH because though this is currently based on a DCE, a preference-based method is preferred.

In conclusion, due to fewer BTH events and the lower administration frequency, ravulizumab is cost-saving compared with eculizumab while increasing the QoL of patients. Therefore, ravulizumab is a beneficial substitute for eculizumab for newly diagnosed adult patients with PNH and patients currently treated with eculizumab.

### Supplementary Information

Below is the link to the electronic supplementary material.

Supplementary file1 (DOCX 108 KB)

Supplementary file2 (DOCX 99 KB)

## Data Availability

Most data are included in the manuscript and its supporting information files. The analyses were largely conducted based on publicly available information which is presented and referenced in the article and supplementary information files. Some of the data generated during and/or analyzed during the current study are not publicly available but are available from the corresponding author on request.
