# Supplementary material for: Cost-effectiveness of ravulizumab compared with eculizumab for the treatment of paroxysmal nocturnal hemoglobinuria in the Netherlands
Source: Eur J Health Econ. 2023 Jan 12;24(9):1455–72. doi: 10.1007/s10198-022-01556-5 (PMC10550878; doi:10.1007/s10198-022-01556-5)
Supplement: Supplementary file 1 — Supplementary file1 (DOCX 108 KB) [file 10198_2022_1556_MOESM1_ESM.docx]

**Appendix 2**

**Figure S1. Tornado diagram illustrating the impact on the incremental costs from the univariate sensitivity analysis for cohort 1.**

**Abbreviations**: BTH: breakthrough hemolysis; Hx: history; IncC5Inhib: incomplete C5 inhibition; trans. prob.: transition probability.

**Figure S2. Tornado diagram illustrating the impact on the incremental QALYs from the univariate sensitivity analysis for cohort 1.**

**Abbreviations:** BTH: breakthrough hemolysis; CAC: complement-amplifying condition; Hx: history; IncC5Inhib: incomplete C5 inhibition; trans. prob.: transition probability.

**Figure S3. Tornado diagram illustrating the impact on the incremental costs from the univariate sensitivity analysis for cohort 2.**

**Abbreviations:** BTH: breakthrough hemolysis; CAC: complement-amplifying condition; Hx: history; IncC5Inhib: incomplete C5 inhibition; trans. prob.: transition probability.

**Figure S4.** **Tornado diagram illustrating the impact on the incremental QALYs from the univariate sensitivity analysis for cohort 2.**

**Abbreviations:** BTH: breakthrough hemolysis; CAC: complement-amplifying condition; Hx: history; IncC5Inhib: incomplete C5 inhibition; trans. prob.: transition probability.

**Figure S5. Cost-effectiveness plane for cohort 1. The red mark reflects the base case ICUR.**

**Abbreviation:** QALYs: quality-adjusted life years.

**Figure S6. Cost-effectiveness plane for cohort 2. The red mark reflects the base case ICUR.**

**Abbreviation:** QALYs: quality-adjusted life years.

**Figure S7. Cost-effectiveness acceptability curve for cohort 1. The red mark represents the probability of being cost-effective at a cost-utility threshold of €20,000 per QALY.**

**Figure S8.** **Cost-effectiveness acceptability curve for cohort 2. The red mark represents the probability of being cost-effective at a cost-utility threshold of €20,000 per QALY.**
