# Supplementary material for: Cost-effectiveness of ravulizumab compared with eculizumab for the treatment of paroxysmal nocturnal hemoglobinuria in the Netherlands
Source: Eur J Health Econ. 2023 Jan 12;24(9):1455–72. doi: 10.1007/s10198-022-01556-5 (PMC10550878; doi:10.1007/s10198-022-01556-5)
Supplement: Supplementary file 2 — Supplementary file2 (DOCX 99 KB) [file 10198_2022_1556_MOESM2_ESM.docx]

# **Appendix 1**

**Table S1. Overview of the model assumptions made for the base case analysis [1-7]**

|  | Assumption | Source |
| --- | --- | --- |
| 1. | In the base case analysis, no patients started at baseline in cohort 3 (eculizumab experienced and up-dosed). | This assumption was made given that comparatively less evidence was available on switching eculizumab up-dosed patients to ravulizumab to inform cohort 3 outcomes. However, data from patients who experienced IncC5Inhib BTH while receiving eculizumab in the first 26 weeks of clinical studies 301 and 302 then switched to ravulizumab between week 26 and 52 reflect that no IncC5Inhib BTH was experienced while receiving ravulizumab [1, 2].  Consequently, given their shared history of eculizumab therapy, patients in cohort 3 were assumed to experience the same outcomes as cohort 2 patients, with the exception of IncC5Inhib BTH events. Model results for cohort 3 were tested as a scenario analysis. |
| 2. | After 26 weeks from baseline, patients in cohort 1 who had not yet experienced IncC5Inhib BTH faced the IncC5Inhib BTH risk of patients in study 302. | This was consistent with the change in profile of patients in cohort 1 by 26 weeks from baseline, as patients recruited for study Patients in study 302 had been stable on treatment with eculizumab for at least 6 months prior to the start of the study [2]. |
| 3. | For the duration of IncC5Inhib BTH events it was assumed that symptoms were experienced for two days out of a fourteen-day cycle. | This was based on the findings from Kelly (2008) and Brodsky (2014) [3, 4]. |
| 4. | BTH events reported as having ’undetermined’ cause in clinical studies 301 and 302 were assumed to represent CAC BTH events and treated as such in the CUA. | In data collected as part of the 301 and 302 studies, ’undetermined’ BTH events could be confirmed to not have elevated free-C5 levels but did not report a concomitant CAC [1, 2]. Therefore, clinical experts were confident that these events were not IncC5Inhib BTH events; however, experts indicated that it was possible that the etiology of the BTH event was not adequately captured, so a CAC-related cause could not be precluded. |
| 5. | In the base-case analysis of the CUA it was assumed that IncC5Inhib BTH events were treated with continuous up-dosing after the second event, while CAC BTH events were not treated with up-dosing. | Based on the treatment recommendations from the Dutch PNH guideline [5]. |
| 6. | The health utility for patients experiencing spontaneous remission was assumed to be the highest utility estimate based on clinical studies 301 and 302 (i.e., omitting any decrements related to BTH and transfusions), plus the increment associated with reduced treatment burden (as no treatment burden is experienced). | This was based on the assumption that patients that experienced a spontaneous remission could discontinue treatment with eculizumab/ravulizumab and remained free from any complications from PNH. See assumption 10. |
| 8. | Continuous up-dosing would not occur for ravulizumab patients. | There were no cases of IncC5Inhib BTH events recorded in clinical trials studies 301 and 302 [1, 2]. |
| 9. | Long-term treatment with ravulizumab would have the same effect on the mortality rate and, therefore, no permanent excess mortality risk is modelled for patients who were stable on treatment with either ravulizumab or eculizumab. | There was no long-term data yet on patients that were treated with ravulizumab at this time. However, given the non-inferior results, compared to eculizumab, in the primary evaluation and extension period in studies 301 and 302, it was expected that long-term results of ravulizumab will also be comparable to eculizumab [1, 2]. |
| 10. | Those who achieve spontaneous remission will stop PNH-related treatment (including complement inhibitor therapy) and the rate of spontaneous submission was assumed to be the same for patients treated with ravulizumab and eculizumab. | Hillmen et al. (1995) state that eculizumab treatment can be discontinued in case of a spontaneous remission. This is assumed to also be the case for ravulizumab [6].  The rate of spontaneous remission was assumed to be equal for patients treated with either complement-inhibitor as spontaneous remission was found irrespective of treatment with ravulizumab and eculizumab and both treatments were not curative. |
| 11. | For the patients in the health state ‘Continuous up-dose of eculizumab’ it was assumed that they cannot experience any further IncC5Inhib BTH events, only CAC BTH events after being up-dosed to a 1200 mg vial. | The Dutch guideline for PNH state an increase of one vial per injection of eculizumab as an option for treating BTH when up-dosing is considered [5]. Since no further increase of dosage is mentioned, it was assumed that this does not occur in practice because the additional vial leads to sufficient C5-inhibition. |
| 12. | While multiple units of pRBC may be required per transfusion, only one transfusion procedure would occur in a cycle of the CUA. | Kelly (2008) noted that hemolysis elevation predominantly occurs around one to two days before the end of each fourteen-day dosing interval [5]. |
| 13. | Administration costs were assumed to be a one-time cost per treatment regardless of the number of vials required during the treatment episode. | Technical assumption to avoid making the model overly complicated. |
| 14. | The price of ravulizumab administration was equal to the administration costs of galsulfase. | To reflect the anticipated longer administration time of ravulizumab versus eculizumab and as ravulizumab administration does not have its own declaration code yet, the administration costs for ravulizumab were based on galsulfase This is an intravenous injection over four hours time. |
| 15. | 50% of the patients we assumed to travel by public transport and 50% use their own car. | The official percentage of people traveling by their own car is 70% in the Netherlands. However, given the context of the travel (i.e. disease treatment or an infusion of 2 to 3 hours) we assumed this to be 20% lower [7]. |

**Abbreviations:** BTH: breakthrough hemolysis; CAC: complement amplifying condition; CUA: cost-utility analysis; IncC5Inhib: incomplete C5 inhibition; PNH: paroxysmal nocturnal hemoglobinuria; pRBC: packed red blood cells.

**Table S2. Overview of calculations made for the patient distribution amongst cohorts in base-case [8-10]**

|  | Number/percentage of patients in 2020 | Calculation | Source |
| --- | --- | --- | --- |
|  |  |  |  |
| Population the Netherlands | 17,426,507 |  | [8] |
| 15 year prevalence PNH | 0.00159% |  | [9] |
| Number of PNH patients in the Netherlands | 277 | Prevalence * Population size |  |
| Percentage of patients diagnosed in the Netherlands | 50% |  | [10] |
| Number of diagnosed patients | 139 | Percentage of patients diagnosed * Number of PNH patients in the Netherlands |  |
| Percentage of patients treated | 60% |  | [10] |
| Number of patients treated | 84 | Number of patients diagnosed * Percentage of patients treated |  |
| Incidence PNH | 0.00013% |  | [9] |
| New patients (cohort 1) (n,%) | ***7, 8.3%*** | Incidence *Percentage of patients treated * Percentage of patients diagnosed * Population in the Netherlands |  |
| Currently treated patients (cohort 2) (n,%) | ***77, 91.6%*** | Number of patients treated – Number of new patients |  |

**Abbreviation**: PNH: paroxysmal nocturnal hemoglobinuria.

**Table S3. Description of the transition probability calculations [1, 2].**

| Transition matrices were constructed in three steps:   1. First, patient-visit-level data for both clinical studies 301 and the 302 were organized to determine the probability of an initial IncC5Inhib BTH event or a CAC BTH event (including ’undetermined’ adjudications) for patients without a history of IncC5Inhib BTH events [1, 2].    - The sample was first restricted to patients without a history of IncC5Inhib BTH events.    - Once a patient had an IncC5Inhib BTH event, subsequent observations for that patient were not included in the analysis. 2. Based on the subset of data identified in the previous step, a full information maximum likelihood multinomial logit model was estimated to predict the outcome state conditional on the initial state (’No BTH’).    - This approach was used to account for censoring from lack of follow-up as well as simultaneous competing risks of transitions to other states.    - Adjusted models controlled for treatment arm and time between initial and follow-up visits.    - Model estimation produced a transition equation for each (initial state–follow-up state) pair that relates the predictors to the probability of transitioning via the estimated coefficients. 3. In the final step, the transition equations developed in the last step were used to calculate the mean transition probabilities for each (initial state-follow-up state) pair.    - This involved multiplying a vector of covariate values by the corresponding vector of estimated coefficients at the observation level for all possible outcomes from the initial state (’No BTH’), applying the formula for calculating predicted probabilities from a multinomial logit to the products, then calculating the mean predicted probabilities across observations.    - In this calculation, the time-between-visits covariate was held constant at a value of 14 days in order to generate two-weekly transition probabilities aligning with the cycle length of the CUA.   Transition probabilities were calculated for both values of the second covariate, which was a binary indicator for whether the patient received ravulizumab or eculizumab in the randomized treatment period (i.e., the first 26 weeks) of the clinical study.  Transition matrices for subsequent IncC5Inhib BTH events were determined in the same manner as for the initial IncC5Inhib and CAC BTH event transitions, with the following exceptions:   - To determine the likelihood of subsequent IncC5Inhib BTH events, the sample was restricted to only patients with a history of IncC5Inhib BTH events. - Only observations that occurred after the first IncC5Inhib BTH event were included in the estimation.   - Note that this selection criteria substantially limited the sample for the 302 clinical study, and thus could only be estimated for the 301 clinical study data. - Since no patient in the ravulizumab arm of either clinical study experienced an IncC5Inhib BTH event, the estimation was only performed for patients in the eculizumab arm. - Finally, this estimation allowed for two initial states, either ’No BTH’ or ’IncC5Inhib BTH’, and observed the subsequent health states from either of these starting states. |
| --- |

**Abbreviations:** BTH: breakthrough hemolysis; CAC: complement-amplifying conditions; CUA: cost-utility analysis; IncC5Inhib: incomplete C5 inhibition.

**Table S4. Description of the calculation of the probability of transfusion and units of packed RBC per transfusion.**

| Estimation of probability of transfusion  The probability that a patient would require a transfusion in a particular cycle of the CUA was estimated separately for periods where a BTH event had occurred and periods where a BTH event had not occurred since the last visit. First, counts of visits at which a BTH event had been experienced since the previous visit and visits where a BTH event had not been experienced since the previous visit were calculated by treatment arm and clinical study. Second, the number of these visits at which a patient had met protocol-specified guidelines for transfusion since the previous visit and/or had received a transfusion of pRBC or WB since the previous visit was determined. The second count was then divided by the first to estimate probabilities.  Estimation of units of packed RBC per transfusion  To estimate the mean number of units of pRBC required per transfusion, data from patient visits at which a transfusion had been received since the previous visit were recorded. For visits at which a patient had met protocol specified guidelines for transfusion since the last visit but no units of pRBC or WBC were recorded, one unit was assumed. The mean and standard deviation across visits at which transfusion guidelines had been met or units had been recorded were then calculated. Standard errors were calculated based on the standard deviation and the number of relevant events. |
| --- |

**Abbreviations:** BTH: breakthrough hemolysis; CUA: cost-utility analysis; pRBC: packed red blood cells; WBC: white blood cells.

**Table S5. Description of the performance of the regression models used to correct the mapped EQ-5D utilities for covariates [1, 2].**

| The mapped EQ-5D data were used to fit separate mixed-effects regression models describing the associations between a number of independent variables and the mapped EQ-5D utilities. Mixed-effects models were employed to better account for the heterogeneity in the panel data used from the 301 and 302 clinical trials – namely due to heterogeneity between individuals in the data and within individuals (e.g., time-varying variables such as time since the last transfusion [1, 2].  Initially, all outcomes of interest were considered as potentially relevant covariates for the regression specification. However, simplifying steps were taken: BTH events were not separated by type, owing to the fact that the limited number of events that occurred in the trials was further reduced by the restriction of observations to the visits when the EORTC was collected (Screening, Day 1, Day 8, Day 29, Day 71, Day 127, and Day 183). As BTH events could not have been observed at Screening or Day 1, given that the BTH event had to have occurred since last visit, only 5 visits remained for observation of BTH event since last visit and EORTC.  This reduced counts of observations with BTH event since last visit and EORTC to:  Study 301: one IncC5Inhib (eculizumab) and three CAC (one eculizumab, two ravulizumab)  Study 302: two IncC5Inhib (eculizumab) and one CAC (eculizumab)  A treatment indicator was omitted because the utility increment of ravulizumab versus eculizumab was based on the DCE survey. In addition to covariates for BTH events and transfusions, a linear count of visits for each patient at each time point was analyzed in the regression model. The count of visits was analyzed to explore a time trend since the start of the trial.  Four additional models were estimated: one each for study 301 and 302, including the treatment indicator, and two models pooling data from study 301 and 302: one including the treatment indicator, and one omitting the treatment indicator. Non-pooled outcomes of study 301 and 302 omitting the treatment indicator were included in the base-case. Non-pooled outcomes of study 301 and 302 with treatment indicator were included in the scenario analysis. The results of the regression analyses can be found in Table S5.  For the BTH health states, it was specified that up-dosing offsets the utility decrement. As the model is specified such that continuous up-dosing occurs on the second IncC5Inhib BTH event, the utility of the initial IncC5Inhib BTH state reflected the scaled disutility, but the utility of the subsequent IncC5Inhib BTH state did not, as the subsequent event was directly treated with up-dosing. The transfusion decrement was applied related to the average number of transfusions the patient in a health state receives. |
| --- |

**Abbreviations:** BTH: breakthrough hemolysis; CAC: complement-amplifying conditions; CUA: cost-utility analysis; EORTC: European Organization for Research and Treatment of Cancer; EQ-5D: EuroQol-5 dimensions- 5 level; IncC5Inhib: incomplete C5 inhibition.

**Table S6. Coefficients from the regression models predicting mapped EQ-5D utilities from BTH, treatment arm (ravulizumab vs eculizumab), transfusion, and visit count [1, 2].**

|  | **Utility** | **95% CI** | | **Source** |
| --- | --- | --- | --- | --- |
| **Study 301 (without tx indicator)** | | | | Study 301 [1] |
| Constant | 0.8084 | 0.7918 | 0.8250 |  |
| BTH-decrement | -0.0576 | -0.1303 | 0.0152 |  |
| Transfusion decrement | -0.0676 | -0.0947 | -0.0405 |  |
| Visit count | 0.0199 | 0.0169 | 0.0230 |  |
| **Study 302 (without tx indicator)** |  |  |  | Study 302 [2] |
| Constant | 0.8875 | 0.8670 | 0.9079 |  |
| BTH-decrement | -0.2850 | -0.3878 | -0.1822 |  |
| Transfusion decrement | -0.0795 | -0.1192 | -0.0397 |  |
| Visit count | 0.0021 | -0.0005 | 0.0047 |  |
| **Study 301 (with tx indicator)** | | | | Study 301 [1] |
| Constant | 0.8117 | 0.7907 | 0.8326 |  |
| BTH-decrement | -0.0576 | -0.1303 | 0.0152 |  |
| Ravulizumab arm (vs. eculizumab) | -0.0066 | -0.0325 | 0.0193 |  |
| Transfusion decrement | -0.0674 | -0.0944 | -0.0403 |  |
| Visit count | 0.0199 | 0.0169 | 0.0230 |  |
| **Study 302 (with tx indicator)** | | | | Study 302 [2] |
| Constant | 0.8734 | 0.8460 | 0.9007 |  |
| BTH-decrement | -0.2832 | -0.3860 | -0.1804 |  |
| Ravulizumab arm (vs. eculizumab) | 0.0284 | -0.0083 | 0.0651 |  |
| Transfusion decrement | -0.0795 | -0.1193 | -0.0398 |  |
| Visit count | 0.0021 | -0.0005 | 0.0047 |  |
| **Pooled (without tx indicator)** | | | | Study 301/ Study 302 [1, 2] |
| Constant | 0.8357 | 0.8194 | 0.8519 |  |
| BTH-decrement | -0.1096 | -0.1686 | -0.0507 |  |
| Transfusion decrement | -0.0634 | -0.0856 | -0.0413 |  |
| Visit count | 0.0121 | 0.0100 | 0.0142 |  |
| Study 302 | 0.0163 | -0.0056 | 0.0382 |  |
| **Pooled (with tx indicator)** | | | | Study 301/ Study 302 [1, 2] |
| Constant | 0.8311 | 0.8117 | 0.8505 |  |
| BTH-decrement | -0.1094 | -0.1683 | -0.0504 |  |
| Ravulizumab arm (vs. eculizumab) | 0.0093 | -0.0124 | 0.0311 |  |
| Transfusion decrement | -0.0636 | -0.0858 | -0.0414 |  |
| Visit count | 0.0121 | 0.0100 | 0.0142 |  |
| Study 302 | 0.0163 | -0.0057 | 0.0382 |  |

**Abbreviations**: BTH: breakthrough hemolysis; CI: confidence interval; tx: treatment.

**Note.** BTH: BTH event experienced since last visit; Transfusion: protocol guidelines for transfusion met since last visit; Tx indicator: indicator whether treated with ravulizumab or eculizumab; visit count: number of visits since start of the trial (time trend).

**Table S7. Overview of the sample and methods used for performing and analyzing the outcomes of the discrete choice experiment.**

| *Methods*  A DCE was designed to understand the importance of different aspects of treatments for PNH and associated outcomes. These “attributes” included (1) life expectancy (2) treatment administration (3) severity and frequency of hemolysis (4) risk of meningococcal infection, and (5) frequency of blood transfusions. Variations of these attributes were combined using a published orthogonal array into choice sets presented in 16 questions, and administered as an online survey to a sample of N = 330 respondents in the Netherlands (broadly representative of the general population in terms of age, gender, ethnicity, and geography). A recruitment panel was used to survey the general public in the Netherlands. The survey was completed online and participants provided consent. The mixed logit model was used to estimate the strength of preference for the attributes in each country. MRS were estimated (for significant attribute levels) by contrasting the coefficients from the regression analysis. MRS indicate the number of units of one attribute that would compensate for the loss of one unit of another attribute.  *Sample: the Netherlands*  The full sample of the Netherlands comprised 502 respondents. The average age of the respondents was 46.7. The distribution of male and female was 45.8% and 54.2%. Most respondents had a paid employment (63.9%) or were not working because of health problems (12.4%). Most respondents were normally fit and well (59.1%), 38.5% of the respondents suffered from a long-term illness, 3.0% was diagnosed with a rare disease, and 5.8% had a family member diagnosed with a rare disease. 68.5% of the respondent lived ten km or less from the nearest hospital, only 2% lived over 50 km from the nearest hospital. The survey included two simple consistency choice tests (dominant choice test and repeated choice test). Response checks showed that 172 respondents failed either the dominant choice test or repeated choice test (or both). The results of the remaining 330 respondents are reported in table 7. |
| --- |

**Abbreviations:** DCE: discrete choice experiment; MRS: Marginal rates of substitution; PNH: paroxysmal nocturnal hemoglobinuria.

**Table S8. Results of mixed logit model of patient preference and the corresponding estimated marginal utilities (expressed as a increment) for differences in attribute levels: sample which passed both logic tests (N=330).**

| **Attributes and levels** | **Coefficients** | **SE** | **z** | **P>\|z\|** | **CI** | | **OR** | **p-value** | | **CI** | |
| --- | --- | --- | --- | --- | --- | --- | --- | --- | --- | --- | --- |
| **Treatment administrations (reference: 6-7 times a year at home, takes 3 hours)** | | | | | | | | | | | |
| 26 times a year at home, takes 1 hour | -0.936 | 0.112 | -8.360 | 0.000 | -1.155 | -0.716 | 0.392 | 0.000 | | 0.315 | 0.488 |
|  | **Passed both logic tests (N=330)** | | | | | **MRS** | | | **Increment** | | |
| **Treatment administration**  Patient receives an infusion every 8 weeks which takes 3 hours (compared with an infusion every 2 weeks which takes 1 hour) | | | | | | 2.453 | | | 0.070 | | |

**Abbreviations**: CI: confidence interval; MRS: marginal rates of substitution; OR: odds ratio; RBC: red blood cell; SE: standard error.

**Note**: Model statistics: Log likelihood = -1970.6; Prob > chi2 <0.00; Number of respondents = 330; Number of choice sets per respondent = 17.

**Table S9. Ravulizumab weight-based dosing regimen for adults [8, 10].**

| **Body weight (kg)** | **Loading dose ravulizumab (mg)** | **Maintenance dose ravulizumab (mg)** | **Initial dose eculizumab (mg)** | **Maintenance dose eculizumab (mg)** |
| --- | --- | --- | --- | --- |
| ≥ 40 to < 60 | 2,400 | 3,000 | 600 | 900 |
| ≥ 60 to < 100 | 2,700 | 3,300 |  |  |
| ≥ 100 | 3,000 | 3,600 |  |  |

**Table S10.** **Modelled medical resource utilization and unit costs for the management of BTH events [3,11-13].**

|  | IncC5Inhib BTH | | CAC BTH | |  |  |
| --- | --- | --- | --- | --- | --- | --- |
| Resource | **Patients using resource (%)** | **Number of units** | **Patients using resource (%)** | **Number of units** | **Unit cost (2019 EUR)** | **Source** |
| General ward admission (day) | 15% | 1 | 23% | 3 | €678.99 | Tomazos et al [11] Cost manual^a^ [12] |
| Intensive care admission (day) | 1% | 1 | 1% | 1 | €2,151.20 | Tomazos et al [11] Cost manual^a^ [12] |
| Dialysis period of 4 weeks | 4% | 1 | 4% | 1 | €5,955.00 | Tomazos et al [11] NZa tariffs ^cd^ [13] |
| Hematology specialist visit | 100% | 1 | 100% | 1 | €140.92 | PNH guideline [3] Cost manual ^e^ [12] |

**Abbreviations**: BTH: breakthrough hemolysis; CAC: complement amplifying condition; IncC5Inhib: incomplete C5 inhibition.

^a^ Based on a nursing day for haemato-oncology (incl. Diagnostics and medication)

^b^ Based on an intensive care nursing day (incl. diagnostics and medication)

^c^ Based on code 140301009: Filtering the blood by an artificial kidney (dialysis) with 4 or 5 dialyses per week in sudden kidney failure, 2nd week

^d^ Based on code 140301009: Filtering the blood by an artificial kidney (dialysis) with 1 to a maximum of 3 dialyses per week in sudden kidney failure

^e^ Based on a polyclinical visits for haemato-oncology

**Table S11.** **Parameter limits used in univariate and probabilistic sensitivity analyses [1,2, 12, 14-16].**

| **Parameter** | **Distribution** | **Mean Value** | **Lower Bound** | **Upper Bound** | **Source** |
| --- | --- | --- | --- | --- | --- |
| **Cohort baseline characteristics** | | | | | |
| Cohort 1 – Age at baseline | Normal | 45.49 | 43.53 | 47.45 | Study 301/ Study 302 [1, 2] |
| Cohort 1 – Proportion female | Beta | 0.46 | 0.24 | 0.68 | Study 301/ Study 302 [1, 2] |
| Cohort 2 – Age at baseline | Normal | 47.71 | 45.71 | 49.70 | Study 301/ Study 302 [1, 2] |
| Cohort 2 – Proportion female | Beta | 0.50 | 0.26 | 0.74 | Study 301/ Study 302 [1, 2] |
| **Transition probabilities eculizumab** | | | | | |
| Cohort 1 eculizumab – BTH Trans. prob.: NoHxIncC5Inhib to IncC5Inhib | Beta | 0.0031 | 0.0063 | 0.0010 | Study 301 [1] |
| Cohort 1 eculizumab – BTH Trans. prob.: NoHxIncC5Inhib to CAC | Beta | 0.0054 | 0.0024 | 0.0095 | Study 301 [1] |
| Cohort 1 eculizumab – BTH Trans. prob.: HxIncC5Inhib to IncC5Inhib | Beta | 0.1429 | 0.0232 | 0.3452 | Study 301 [1] |
| Cohort 1 eculizumab – BTH Trans. prob.: HxIncC5Inhib_IncC5Inhib to IncC5Inhib | Beta | 0.3333 | 0.1127 | 0.6043 | Study 301 [1] |
| Cohort 2 eculizumab – BTH Trans. prob.: NoHxIncC5Inhib to IncC5Inhib | Beta | 0.0010 | 0.0036 | 0.0000 | Study 302 [2] |
| Cohort 2 eculizumab – BTH Trans. prob.: NoHxIncC5Inhib to CAC | Beta | 0.0031 | 0.0063 | 0.0010 | Study 302 [2] |
| Cohort 2 eculizumab – BTH Trans. prob.: HxIncC5Inhib to IncC5Inhib | Beta | 0.3333 | 0.9852 | 0.0000 | Study 302 [2] |
| Cohort 2 eculizumab – BTH Trans. prob.: HxIncC5Inhib_IncC5Inhib to IncC5Inhib | Beta | 0.3333 | 0.9852 | 0.0000 | Study 302 [2] |
| **Transition probabilities ravulizumab** |  |  |  |  |  |
| Cohort 1 ravulizumab – BTH Trans. prob.: NoHxIncC5Inhib to IncC5Inhib | Beta | 0.0000 | 0.0000 | 0.0000 | Study 301 [1] |
| Cohort 1 ravulizumab – BTH Trans. prob.: NoHxIncC5Inhib to CAC | Beta | 0.0026 | 0.0007 | 0.0058 | Study 301 [1] |
| Cohort 2 ravulizumab – BTH Trans. prob.: NoHxIncC5Inhib to IncC5Inhib | Beta | 0.0000 | 0.0000 | 0.0000 | Study 302 [2] |
| Cohort 2 ravulizumab – BTH Trans. prob.: NoHxIncC5Inhib to CAC | Beta | 0.0001 | 0.0000 | 0.0004 | Study 302 [2] |
| **No BTH: Prob. transfusion in two-week period** | | | | | |
| Cohort 1 eculizumab – No BTH: prob. transfusion in 2wk period | Beta | 0.09 | 0.07 | 0.10 | Study 301/ Study 302 [1, 2] |
| Cohort 2 eculizumab – No BTH: prob. transfusion in 2wk period | Beta | 0.02 | 0.01 | 0.03 | Study 301/ Study 302 [1, 2] |
| Cohort 1 ravulizumab – No BTH: prob. transfusion in 2wk period | Beta | 0.06 | 0.05 | 0.07 | Study 301/ Study 302 [1, 2] |
| Cohort 2 ravulizumab – No BTH: prob. transfusion in 2wk period | Beta | 0.02 | 0.02 | 0.03 | Study 301/ Study 302 [1, 2] |
| **No BTH: Units of pRBC per transfusion** | | | | | |
| Cohort 1 eculizumab – No BTH: units of pRBC per transfusion | Gamma | 1.59 | 1.46 | 1.73 | Study 301/ Study 302 [1, 2] |
| Cohort 2 eculizumab – No BTH: units of pRBC per transfusion | Gamma | 1.57 | 1.35 | 1.81 | Study 301/ Study 302 [1, 2] |
| Cohort 1 ravulizumab – No BTH: units of pRBC per transfusion | Gamma | 1.68 | 1.51 | 1.86 | Study 301/ Study 302 [1, 2] |
| Cohort 2 ravulizumab – No BTH: units of pRBC per transfusion | Gamma | 1.58 | 1.34 | 1.83 | Study 301/ Study 302 [1, 2] |
| **With BTH: Prob. transfusion in two-week period** | | | | | |
| Cohort 1 eculizumab – BTH: prob. transfusion in 2wk period | Gamma | 0.30 | 14.00 | 14.00 | Study 301/ Study 302 [1, 2] |
| Cohort 2 eculizumab – BTH: prob. transfusion in 2wk period | Gamma | 0.38 | 2.03 | 14.00 | Study 301/ Study 302 [1, 2] |
| Cohort 1 ravulizumab – BTH: prob. transfusion in 2wk period | Gamma | 0.17 | 1.76 | 14.00 | Study 301/ Study 302 [1, 2] |
| Cohort 2 ravulizumab – BTH: prob. transfusion in 2wk period | Gamma | 0.00 | 0.00 | 0.00 | Study 301/ Study 302 [1, 2] |
| **With BTH: Units of pRBC per transfusion** | | | | | |
| Cohort 1 eculizumab – BTH: units of pRBC per transfusion | Gamma | 1.83 | 1.52 | 2.17 | Study 301/ Study 302 [1, 2] |
| Cohort 2 eculizumab – BTH: units of pRBC per transfusion | Gamma | 2.33 | 0.94 | 4.35 | Study 301/ Study 302 [1, 2] |
| Cohort 1 ravulizumab – BTH: units of pRBC per transfusion | Gamma | 1.50 | 0.69 | 2.63 | Study 301/ Study 302 [1, 2] |
| Cohort 2 ravulizumab – BTH: units of pRBC per transfusion | Gamma | 0.00 | 0.00 | 0.00 | Study 301/ Study 302 [1, 2] |
| **No-BTH-event health utilities** | | | | | |
| Cohort 1 eculizumab – No BTH-event health utility | Beta | 0.87 | 0.85 | 0.89 | Study 301/ Study 302 [1, 2] |
| Cohort 1 ravulizumab – No BTH-event health utility | Beta | 0.87 | 0.85 | 0.87 | Study 301/ Study 302 [1, 2] |
| Cohort 2 eculizumab – No BTH-event health utility | Beta | 0.89 | 0.87 | 0.91 | Study 301/ Study 302 [1, 2] |
| Cohort 2 ravulizumab – No BTH-event health utility | Beta | 0.89 | 0.87 | 0.91 | Study 301/ Study 302 [1, 2] |
| **BTH-event health-utility decrement** | | | | | |
| Cohort 1 – BTH-event utility decrement | Gamma | -0.06 | 0.01 | 0.15 | Study 301/ Study 302 [1, 2] |
| Cohort 2 – BTH-event utility decrement | Gamma | -0.29 | 0.19 | 0.40 | Study 301/ Study 302 [1, 2] |
| Persistence of IncC5Inhib BTH events – user-specified probability | Gamma | 0.50 | 0.29 | 0.77 | Study 301/ Study 302 [1, 2] |
| **Transfusion health-utility decrement** | | | | | |
| Cohort 1 – Transfusion utility decrement | Gamma | -0.07 | 0.04 | 0.10 | Study 301/ Study 302 [1, 2] |
| Cohort 2 – Transfusion utility decrement | Gamma | -0.08 | 0.04 | 0.12 | Study 301/ Study 302 [1, 2] |
| Convenience health-utility benefit | Gamma | 0.07 | 0.07 | 0.07 | DCE (table S7, S8, S8) |
| **Costs** | | | | | |
| Vaccine cost | Gamma | 142.42 | 81.41 | 220.22 | Medicijnkosten.nl [14] |
| Administration cost eculizumab | Gamma | 189.29 | 104.20 | 281.87 | NZa reference cost [15] |
| Administration cost ravulizumab | Gamma | 334.22 | 191.03 | 516.79 | NZa reference cost [15] |
| Transfusion administration cost | Gamma | 225.26 | 128.76 | 348.31 | Dutch cost manual[12] |
| pRBC unit cost | Gamma | 230.60 | 131.81 | 356.57 | Dutch cost manual[12] |
| Parking costs (per visit) | Gamma | 3.00 | 4.64 | 1.72 | Dutch cost manual[12] |
| Cost per kilometer | Gamma | 0.19 | 0.29 | 0.11 | Dutch cost manual[12] |
| Cost of productivity (per hour) | Gamma | 36.88 | 57.02 | 21.08 | Dutch cost manual[12] |
| **Other** | | | | | |
| Percentage of patients traveling by personal transport (%) | Normal | 50 | 26 | 74 | Dutch cost manual[12] |
| Distance to expert center (to hospital and back) in km | Normal | 147 | 75 | 219 | Dutch cost manual[12] |
| Distance to hospital (to hospital and back) in km | Normal | 14 | 7.1 | 20.9 | Dutch cost manual[12] |
| Average labor participation rate (45-75 years) | Normal | 0.55 | 0.3 | 0.8 | CBS [16] |

**Abbreviations**: BTH: breakthrough hemolysis; CAC: complement amplifying condition; DCE: discrete choice experiment; Hx: history of; IncC5Inhib: incomplete C5 inhibition; NZa: Dutch Healthcare Authority; pRBC: packed red blood cell; Trans. Prob.: transition probability.

**Table S12. Scenarios included in the cost-utility analysis.**

| Scenario | Base Case |
| --- | --- |
| Time horizon was set to 10 years | 101 years |
| Time horizon was set to 20 years | 101 years |
| Discount rate benefits and costs were set to 0% | Discount rate costs: 4%, Discount rate benefits: 1.5% |
| Discount rate benefits and costs were set to 6% | Discount rate costs: 4%, Discount rate benefits: 1.5% |
| IncC5Inhib BTH duration (days) were set to 1 day | 2 days |
| IncC5Inhib BTH duration (days) were set to 3 days | 2 days |
| IncC5Inhib BTH duration (days) were set to 7 days | 2 days |
| Formulation ravulizumab was set to 100 mg/mL | 10mg/mL |
| Cohort 3 was included in the aggregate population (20% patients started in cohort 3 [17]) | 0% of the patients started in cohort 3^a^ |
| Utility increment of ravulizumab vs. eculizumab was calculated from studies 301 and 302 | Calculated from the DCE |
| The general health-utility cap was excluded | Included |
| EORTC to EQ-5D-3L mapping based on Longworth (2014) [18] | Versteegh (2012) [19] |
| HRQoL regression analysis based on pooled population | Separate for studies 301 and 302 |
| No up-dosing of eculizumab happened after IncC5Inhib BTH events | Continuous up-dosing after second IncC5Inhib BTH event |
| Continuous up-dosing of eculizumab after first IncC5Inhib BTH event | Continuous up-dosing after second IncC5Inhib BTH event |
| BTH excess mortality (HR) vs. background was set to 1.20 | 4.81 |
| BTH excess mortality (HR) vs. background was set to 0 | 4.81 |
| Percentage receiving Alexion-funded homecare was set to 100% | 0% |
| Extrapolation of initial IncC5Inhib BTH risk after 26 weeks was excluded | Included |
| Persistence of IncC5Inhib BTH was user defined 25% | 50% |
| Persistence of IncC5Inhib BTH was user defined 75% | 50% |
| Days in 2-week cycle with IncC5Inhib BTH symptoms was set to 4 | 2 |
| CAC BTH up-dosing was included | Excluded |
| Healthcare payer's perspective was used and societal costs were excluded | Societal perspective |
| Spontaneous remission was excluded | Included and modelled with an annual incidence of 1.59% |

**Abbreviations**: BTH: breakthrough hemolysis; CAC: complement amplifying condition; DCE: discrete choice experiment; EORTC: European Organization for Research and Treatment of Cancer; EQ-5D-3L: EuroQol-5 dimensions-3 level; HR: hazard ratio; HRQoL: Health-Related Quality of Life; Hx: history of; IncC5Inhib: incomplete C5 inhibition.

^a^ Characteristics of patients in cohort 3 were assumed to be the same as those of patients in cohort 2, due to the lack of an clinical trial of up-dosed eculizumab patients switching to ravulizumab.

# **References**

1. Lee, J.W., de Fontbrune, F.S., Lee, L.W.L., Pessoa, V., Gualandro, S., Füreder, W., Ptushkin, V., Rottinghaus, S.T., Volles, L., Shafner, L., Aguzzi, R., Pradhan, R., Schrezenmeier, H., Hill, A.: Ravulizumab (ALXN1210) vs eculizumab in adult patients with PNH naive to complement inhibitors: The 301 study. Blood. 133, 530–539 (2019). https://doi.org/10.1182/blood-2018-09-876136

2. Kulasekararaj, A.G., Hill, A., Rottinghaus, S.T., Langemeijer, S., Wells, R., Gonzalez-Fernandez, F.A., Gaya, A., Lee, J.W., Gutierrez, E.O., Piatek, C.I., Szer, J., Risitano, A., Nakao, S., Bachman, E., Shafner, L., Damokosh, A.I., Ortiz, S., Röth, A., Peffault de Latour, R.: Ravulizumab (ALXN1210) vs eculizumab in C5-inhibitor–experienced adult patients with PNH: The 302 study. Blood. 133, 540–549 (2019). https://doi.org/10.1182/blood-2018-09-876805

3. Kelly, R., Arnold, L., Richards, S., Hill, A., vanBijnen, S., Muus, P., Dorr, D., Brodsky, R., Khursigara, G., Rother, R.P., Hillmen, P.: Modification of the Eculizumab Dose to Successfully Manage Intravascular Breakthrough Hemolysis in Patients with Paroxysmal Nocturnal Hemoglobinuria. Blood. 112, 3441–3441 (2008). https://doi.org/10.1182/blood.v112.11.3441.3441

4. Brodsky, R.A.: Paroxysmal nocturnal hemoglobinuria. Blood. 124, 2804–2811 (2014). https://doi.org/10.1182/blood-2014-02-522128

5. Muus, P., Langemeijer, S., Halkes, S., Zeerleder, S., Schouten, H., Boekhorst te, P., Span, B., Witte de, M., Bartels, M., Jansen, A., Stiene, A., Preijers, F., Pampus van, L., Evers, D., Nijziel, M., Laros, B., Heijden van der, O.: Guideline for Paroxysmal Nocturnal Hemoglobinuria (Dutch: Richtlijn Paroxysmale Nachtelijke Hemoglobinurie). , Nijmegen (2016)

6. Rosse, W.F.: The natural history of paroxysmal nocturnal hemoglobinuria. N. Engl. J. Med. 333(19):12, (1995). https://doi.org/10.1056/NEJM199511093331904

7. Dutch Statistics: How much do we use public transport? (Dutch: Hoeveel wordt er met het openbaar vervoer gereisd?), https://www.cbs.nl/nl-nl/visualisaties/verkeer-en-vervoer/personen/openbaar-vervoer#:~:text=In 2019 legden alle inwoners,bus%2C tram of metro gereisd.

8. Dutch Statistics: Bevolking; kerncijfers

9. Hill, A., Platts, P.J., Smith, A., Richards, S.J., Cullen, M.J., Hill, Q.A., Roman, E., Hillmen, P.: The Incidence and Prevalence of Paroxysmal Nocturnal Hemoglobinuria (PNH) and Survival of Patients in Yorkshire. Blood. 108, 985–985 (2006). https://doi.org/10.1182/blood.v108.11.985.985

10. Alexion internal data PNH register.: No Title.

11. Tomazos, I., Sierra, J.R., Johnston, K.M., Cheung, A., Brodsky, R.A., Weitz, I.C.: Cost burden of breakthrough hemolysis in patients with paroxysmal nocturnal hemoglobinuria receiving ravulizumab versus eculizumab. Hematol. (United Kingdom). 25, 327–334 (2020). https://doi.org/10.1080/16078454.2020.1807226

12. Hakkaart-van Roijen, L., van der Linden, N., Bouwmans, C., Kanters, T., Swan Tan, S.: Cost Manual: Methodology and Reference Prices for Healthcare Economic Evaluations. (Dutch: Kostenhandleiding: Methodologie van kostenonderzoek en referentieprijzen voor economische evaluaties in de gezondheidszorg. Dutch Natl. Heal. Care Inst. 1–73 (2016)

13. Dutch Healthcare Authority (Dutch: Nederlandse Zorgautoriteit) (NZa): Open DIS Data - DBC zorgproductenzoeker, https://www.opendisdata.nl/

14. Dutch Institute National Health Care (Zorginstituut Nederland): Medicijnkosten, https://www.medicijnkosten.nl/

15. Dutch Healthcare Authority (Dutch: Nederlandse Zorgautoriteit) (NZa): Prestatie- en tarieventabel add-on geneesmiddelen (2016-12) -

16. Centraal Bureau voor de Statistiek: StatLine - Life expectancy; sex, age (per year and period of five years) (Dutch: Levensverwachting; geslacht, leeftijd (per jaar en periode van vijf jaren), https://www.cbs.nl/nl-nl/cijfers/detail/37360ned

17. Newton, D.J., McKinley, C.E., Ricardo, A., Hoarty, M.D., Arnold, L., Riley, K., Copeland, N., Munir, T., Griffin, M., Hill, A., Hillmen, P.: Assessment of Eculizumab and C5 Levels in PNH Treatment: Molar Ratio of Eculizumab to C5 Level Effectively Predicts Ongoing Dosage Changes in a Cohort of 50 Patients Treated with Eculizumab. Blood. 130, 3484–3484 (2017). https://doi.org/10.1182/BLOOD.V130.SUPPL_1.3484.3484
